# Nonleukemic granulocytic sarcoma of orbit after blunt trauma: A case report and review of literature

**DOI:** 10.1097/MD.0000000000010373

**Published:** 2018-04-13

**Authors:** Yuan Cheng, Chun’e Yu, Sha Zhu, Linghong Guo, Yi Zhang, Yiwen Zhang, Xuelei Ma

**Affiliations:** State Key Laboratory of Biotherapy and Cancer Center, West China Hospital, Sichuan University and Collaborative Innovation Center, Chengdu, People's Republic of China.

**Keywords:** diagnosis, etiology, nonleukemic granulocytic sarcoma, trauma, treatment

## Abstract

**Rationale:**

Granulocytic sarcoma without invasion of bone marrow or blood is very rare. The diagnosis of it is usually overlooked and the treatment has not reached a consensus. Meanwhile, the onset of this kind of disease is not clear.

**Patient concerns:**

Diagnose patients in early stage and help choose the right treatment strategies.

**Diagnoses:**

The ultimate diagnosis was nonleukemic granulocytic sarcoma after blunt trauma.

**Interventions:**

Surgery was the initial treatment option. Chemotherapy including idarubicin (70 mg, D1–D3) and cytosine arabinoside (100 mg, D1–D7) and radiotherapy of total 3,060 cGy were then administered but failed to control the disease. Hematopoietic stem cell transplantation was finally administered.

**Outcomes:**

No evidence of disease progression or spread according to the latest follow-up.

**Lessons:**

The etiology of nonleukemic granulocytic still remains unclear, though trauma seems to be a potential predisposing factor and deserves more attention for early diagnosis and timely and proper treatment. Systemic chemotherapy is more effective than radiotherapy or surgery. Hematopoietic stem cell transplantation is an alternative choice after the failure of chemotherapy.

## 1 Introduction

Granulocytic sarcoma (GS) has been known as a localized tumor composed of immature myeloid precursor cells, and as a sign of extramedullary infiltration of leukemic especially, in patients with acute myelogenous leukemia (AML). Trauma as a cause of GS is quite rare, and not attracts enough attention because it is a controversial predisposing factor in the initiation of tumor. In our case, we offered a hypothesis for gaining an insight into the connection between trauma and malignancy. Most of the reported cases which had a localized tumor after trauma usually, developed to non-Hodgkin's lymphoma.^[1–4]^ Meanwhile, GS without leukemia, or other myeloproliferative disorders is relatively rare.^[5]^ Because of the differences, like the average age of onset, and prognosis, nonleukemic GS may be a different category from GS appearing during, or after AML. Owing to its rarity, there is still no defined treatment for nonleukemic GS, and the diagnosis is also often overlooked.

Here we reported a rare case of post-trauma nonleukemic GS of the orbital region. After accurate diagnosis, this patient has been under systemic treatment.

## 2 Case report

**Figure 1:**
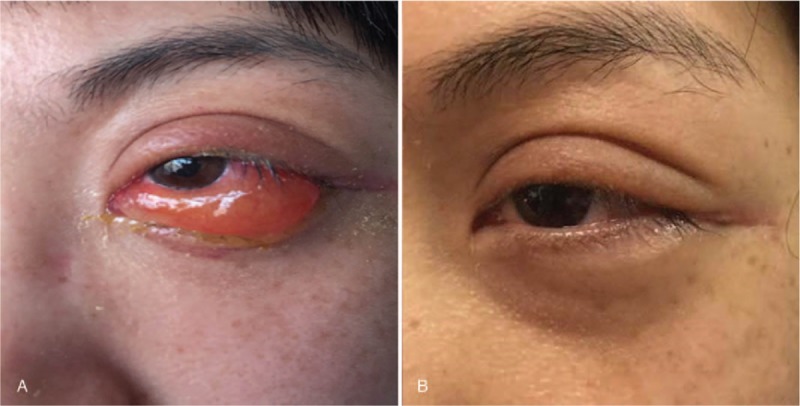
A soft-tissue mass of the patient's left eye before (A) and after (B) surgery.

**Figure 2:**
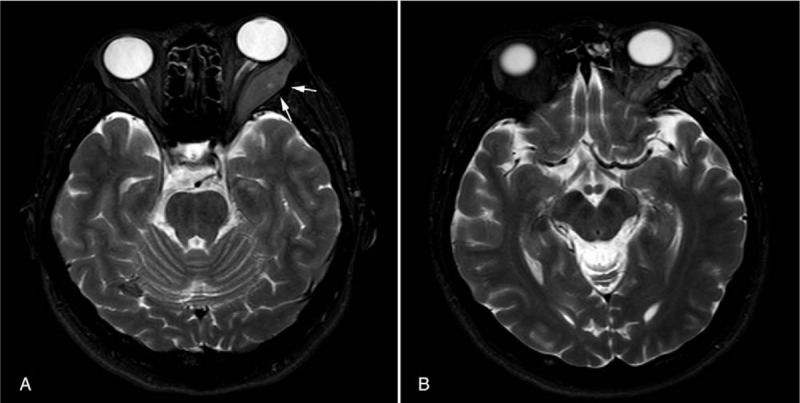
MRI revealed a mass of left lateral oculus. (A) before surgery (the mass is indicated by arrows, 3.6 × 1.3 cm). (B) after surgery. MRI = magnetic resonance imaging.

**Figure 3:**
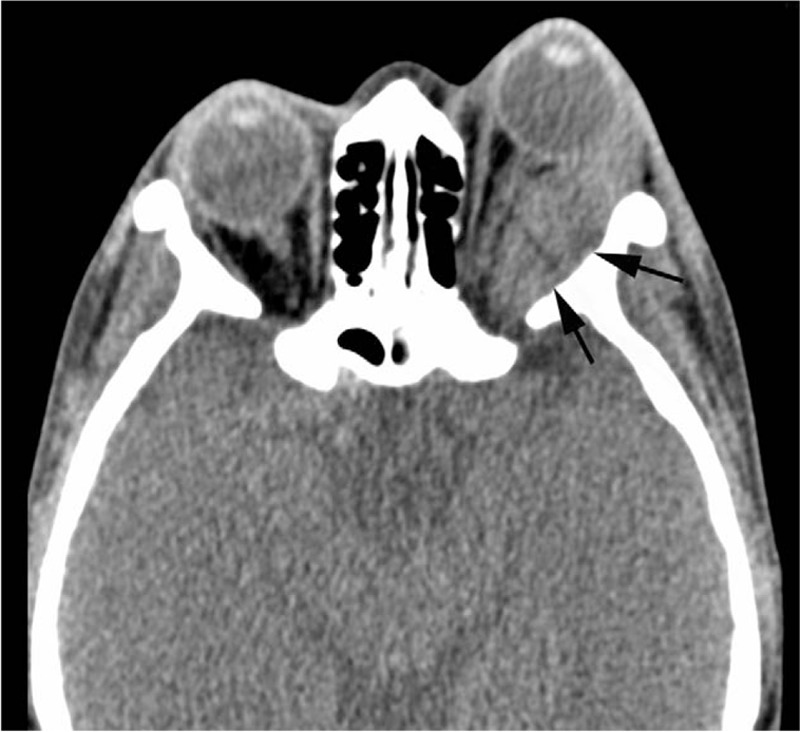
CT scan revealed a larger mass out of the left eye muscular cone space (the mass is indicated by arrows, 4.4 × 1.7 cm). CT = computed tomography.

This study was approved by institutional board of West China Hospital. On admission, this patient already wrote informed consents of offering her case data for scientific research. A 33-year-old woman patient presented in the year July 2016 with a non-resolving soft-tissue mass in left oculus (Fig. 1A). She was once elbowed by a kid at the same place more than 1 month ago, and this mass was noticed after being hit. No complaint of obvious pain, diplopia, or decreased vision was presented. Clinical examination revealed a soft mobile swelling mass which was approximately 2 × 1.5 × 0.5 cm, and the surrounding skin was normal. No notable history of hematopathy, or no other significant signs such as enlarged lymph nodes, or weight loss was presented. Orbital magnetic resonance imaging (MRI) showed no sign of fraction but a 3.6 × 1.3 cm mass of soft-tissue density in left lateral orbital region, and there was vague continuity between the tumor, and the wall of maxillary sinus (Fig. 2A). The lesion was thought to go through a normal change by the accident, so she didn’t receive any special treatment. A follow-up was requested in order to track the change of this mass, and in case of occurrence of other symptoms. One month later, the patient was readmitted with swelling pain, and redness of the left oculus. Orbital computed tomography (CT) of the head, and neck region showed that the mass located out of the left eye muscular cone space had increased in size to 4.4 × 1.7 cm (Fig. 3). But type-B ultrasound showed a hematoma in the left oculus. The results of laboratory examination including blood routine test, electrolytes, and urine analysis were normal. Therefore, we considered these symptoms had been caused by the inflammation response, and swelling of surrounding tissue. After prednisone (30 mg, qd, decrement of 5 mg per week) was administrated, there was symptomatic relief. But after 1 month treatment, the patient developed decreased vision in left eye. According to former imaging examinations, eye-sight loss was thought to be a result of optic nerve compression. Therefore, surgical resection of the mass was performed in the year November 2016 (Fig. 1B).

## 3 Pathological findings

**Figure 4:**
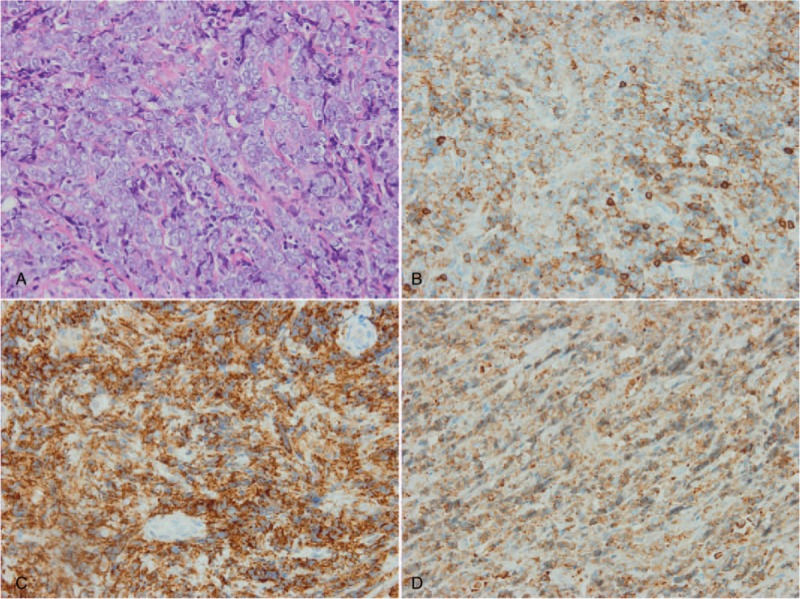
HE and immunohistochemical staining of specimen. (A) HE staining, ×400; (B) LCA staining ×400; (C) CD43, ×400; (D) MPO staining, ×400. CD43 = leukosialin, cluster of differentiation 43, HE = hematoxylin-eosin staining, LCA = leukocyte common antigen, MPO = myeloperoxidase.

The gross pathology revealed that it was a canary yellow color solid mass of dimension 4 × 3.5 × 1 cm. Histopathological diagnosis of the specimen from the surgery indicated GS. Immunohistochemical staining revealed CD43, LCA, MPO, and CD117 were positive whereas PCK, EMA, CD20, CD3, CD30, SALL4, S-100 and HMB45 were negative (Fig. 4), which was consistent with a granulocytic phenotype, confirming the diagnosis of GS. Hematoxylin, and eosin staining of the tissue showed diffuse infiltration of poorly, differentiated cells, with nuclear pleomorphism, and mitotic activity. Bone marrow cytology, and bone marrow biopsy revealed no infiltration of tumor cell. No other hematological, and biochemical parameters of leukemia, or myeloprolifrative disorder were found.

## 4 Treatment and follow-up

The MRI after surgery revealed a smaller mass, and swelling of the surrounding tissue due to the resection surgery. But invasion of infratemporal fossa, and pterygopalatine fossa was also apparent (Fig. 2B). Then she received systemic chemotherapy including idarubicin (70 mg, D1−D3), and cytosine arabinoside (100 mg, D1–D7) which started in the year December, 2016. After 2 cycles of chemotherapy, the mass did not decreased in size. Therefore, she was started on local irradiation for the tumor of total 3,060 cGy with a daily fraction of 180 cGy on the region of left eye during the year February, 2017. However, after completing chemotherapy, and radiotherapy, the subsequent MRI revealed spread of the tumor in left oculus, and diffuse infiltration of surrounding tissue. There was no sign of involvement of blood and bone marrow. Therefore, the third cycle of chemotherapy was started late in the year March 2017 which was aimed to control this disease. After the third cycle of chemotherapy, this patient completed hematopoietic stem cell transplantation in June 2017. Except for some common side effects of chemotherapy drugs, no evidence of disease progression or spread during our follow-up. According to her latest follow-up in March, 2018, she had been infected with virus due to immunosuppressive condition after hematopoietic stem cell transplantation. But routine blood test showed she had been in complete remission.

## 5 Discussion

GS is a kind of solid tumor composed of immature granulocytes, and initially, named Chloroma because of the myeloperoxidase (MPO) in the tumor cells which leads to greenish cut surface.^[6]^ The conception of extramedullary myeloid cell sarcoma (EMT) was mentioned in the year 1988 by Davery, including extramedullary infiltration of leukemia and isolated GS.^[7]^ The latter one is quite rare worldwide, and also known as nonleukemic GS, which means no peripheral blood, or bone marrow involved. Most common sites for GS include lymph nodes, skin, head, spinal cord, soft tissue, and bone.^[8,9]^

Chronic inflammation and scar of injury developing into tumor has been described in a lot of literatures.^[10–12]^ However, trauma still remains a controversial risk factor in the onset of tumor. During our research, reported cases related to lymphoma usually, initiate at head after blunt trauma, such as scalp,^[1]^ oculus, ^[2]^ and uveitis.^[4]^ Blunt trauma can cause local tissue degeneration, and necrosis, and finally, result in cell atypia during the healing process.^[2]^ Almost all of the reported cases were non- Hodgkin's lymphoma, but there was not any case of post-trauma nonleukemic GS. However, there is one special case. A 30-year-old woman who was diagnosed of AML, and got complete remission. Later, she was hit in the forehead, and developed a swelling which was diagnosed of isolated GS without leukemic relapse.^[13]^ Whereas, it is still a big challenge to associate trauma with tumorgenesis. Time intervals between trauma, and tumor, an important factor for associating these 2 issues, differs from each other. For soft tissue sarcoma, it can be 9 years.^[14]^ But for hematological diseases like non-Hodgkin's lymphoma, the development of tumor usually happened within a year.^[1–4]^ If there would be more attention on the association between trauma, and tumorgenesis, early follow-up of the change in the lesion could be achieved. Meanwhile, lack of recognition of this possibility may result in delay of diagnosis and timely treatment.

GS usually, present in acute AML, or just after the onset of AML. Studies also find it could occur in the blastic phase of chronic myelogenous leukemia (CML) or is symptom of myeloid proliferative disease. However, GS as a manifestation before the onset of AML is rather rare.^[5]^ The prognosis of GS is usually, poor, and most of the nonleukemic GS will develop to AML, and finally, cause death. The time interval of untreated nonleukemic GS transforming to AML is usually between 10 to 12 months period.^[15]^ According to a small sample study, the median failure-free-survival (FFS) of nonleukemic GS was 12 months.^[16]^ Whereas there are patients who get remission after appropriate treatment. For instance, 2 cases of GS reported by Garcia^[17]^ had been in remission condition for more than 10 years. Although orbital, and ocular region is one of the most common sites for the extramedullary infiltration location of acute leukemia,^[18]^ so far as we know, there has been no report of nonleukemic GS at this site. The pathogenesis of this kind of disease is still unclear. A study found that almost 54.3% of those cases could be identified by chromosomal aberrations, but specific clinic significance still needs to be discovered.^[19]^

The diagnosis of GS especially, without blood, or bone marrow involved is quite difficult. Nonleukemic GS is initially, often misdiagnosed as lymphoma such as non-Hodgkin lymphoma,^[20]^ especially, when it lacks the clues for the diagnosis of GS, differentiated poorly, and no appearance of green surface.^[21]^ Nieman et al reported that in their study, 48% tumors showed no differentiation of granulocytes, and even got some features similar to lymphoma.^[22]^ GS can be divided into 3 types based on the histological finds: blastic, immature and well-differentiated.^[21]^ Immunohistochemistry (IHC) can increase the accuracy of the diagnosis of GS. MPO is thought to be the most meaningful marker owning to its high specificity and sensitivity of myeloid cells. However, when the cells are less-differentiated, the expression of MPO can be quite low.^[23]^ Some studies suggesting other markers for the diagnosis of GS. For instance, Traweek et al ^[24]^ suggested that lysozyme and CD68 expressed on less-differentiated myeloid cells but not on lymphocytes which could help to distinguish GS from lymphoma. And CD43 can be detected in almost all GS cases. Therefore, CD43, CD68, and MPO can successfully identify 96% of extramedullary myeloid cell tumors, which should be included in the immunohistochemical panel of GS.^[24]^ For this patient in our case, the immunohistochemical staining revealed CD43 and MPO were positive which helped us with the final diagnosis.

The treatment strategies of nonleukemic GS still remain unclear because of the rarity of this disease. So far, systemic chemotherapy has been usually recommended for nonleukemic GS. Imrie et al found that the application of anti-leukemic chemotherapy at the time of diagnosis of GS could reduce the possibility of subsequent leukemia such as AML and prolong the survival of patients.^[25]^ Therefore, to reduce the risk of developing to leukemia, immediate intensive chemotherapy similar to that used to treat AML during nonleukemic period is quite important.^[9]^ For the patient in this case, we administrated both local and systemic treatment. Resection surgery helped to relieve symptoms and make the final diagnosis. Nonleukemic GS is sensitive to radiotherapy, however, the condition of our patient got worse after irradiation which indicated that it was controversial to apply this treatment to nonleukemic GS patients. Meanwhile, studies found that local treatments did not seem to improve the survival, and prognosis, which suggested that isolated GS might also be a systemic disease.^[16]^ This phenomenon indicated that resection and irradiation as local treatment methods are not enough for nonleukemic GS. Stem cell transplantation has also been considered as one method to get CR (complete remission),^[26]^ however, owning to the limited cases, the efficacy of this treatment needs further investigation. In this case, chemotherapy and radiotherapy did not control this disease well. Therefore, this patient received stem cell transplantation, and has been in complete remission phase during 8-month follow-up.

In summary, we presented a rare case of post-traumatic nonleukemic GS in orbital region without blood or bone marrow involved. Trauma can be a potential risk factor of not only lymphoma, and other solid tumors but also GS. Immunohistochemical staining is vital for the early diagnosis of this kind of disease which is usually misdiagnosed as Non-Hodgkin lymphoma. Our treatment shows that resection, and irradiation as local treatment are not effective, and it is essential to administer systemic chemotherapy. After the failure of combined chemotherapy, and radiotherapy, hematopoietic stem cell transplantation can be considered.

## Author contributions

**Conceptualization:** Yuan Cheng, Xuelei Ma, Yiwen Zhang.

**Data curation:** Yuan Cheng, Chun’e Yu, Sha Zhu, Yi Zhang.

**Methodology:** Yuan Cheng.

**Resources:** Chun’e Yu.

**Supervision:** Xuelei Ma, Yiwen Zhang.

**Writing – original draft:** Yuan Cheng.

**Writing – review & editing:** Sha Zhu, Linhong Guo, Xuelei Ma, Yiwen Zhang.
